# Evaluation of Antioxidant Activities of Ampelopsin and Its Protective Effect in Lipopolysaccharide-Induced Oxidative Stress Piglets

**DOI:** 10.1371/journal.pone.0108314

**Published:** 2014-09-30

**Authors:** Xiang Hou, Jingfei Zhang, Hussain Ahmad, Hao Zhang, Ziwei Xu, Tian Wang

**Affiliations:** 1 College of Animal Science and Technology, Nanjing Agricultural University, Nanjing, Jiangsu, P.R. China; 2 Institute of Animal Husbandry and Veterinary Science, Zhejiang Academy of Agricultural Sciences, Hangzhou, Zhejiang, P.R. China; University of Arkansas for Medical Sciences; College of Pharmacy, United States of America

## Abstract

The aim of this study was to investigate the antioxidant potential of ampelopsin (APS) by using various methods *in vitro*, as well as to determine effects of APS on LPS-induced oxidative stress in piglets. The results showed that APS exhibited excellent free radical scavenging by DPPH, ABTS, O_2_•^−^, H_2_O_2_ and ferric reducing antioxidant power. Ampelopsin also protected pig erythrocytes against AAPH-induced apoptosis and hemolysis, decreased total superoxide dismutase activity, and increased lipid peroxidation. Furthermore the results demonstrated that APS enhanced the total antioxidant capacity and decreased the malondialdehyde and protein carbonyl contents in LPS-treated piglets. The results of the present investigation suggest that APS possesses a strong antioxidant activity and alleviates LPS-induced oxidative stress, possibly due to its ability to prevent reactive oxygen species.

## Introduction

Ampelopsin (3,5,7,3′,4′,5′-hexahydroxy-2,3-dihydroflavonol, APS), is the main bioactive component and most common flavonoid (about 20–30% w/w) in dry tender stems and leaves. In particular, APS comprises more than 40% of the flavonoid content in the cataphyll of the plant *Ampelopsis grossedentata (Hand-Mazz) W.T. Wang*. It is known as vine tea, and has been a health beverage in the south of China for thousands of years. Ampelopsin plays a wide variety of health roles, including anti-inflammatory, anti-hypertension, cough relieving, antioxidant, hepatoprotective, antimicrobial, and anticarcinogenic actions [1], [2]. However, to the best of our knowledge, there are so far no systematic reports relating to the antioxidant capacity of APS using both *in vivo* and *in vitro* assays.

Lipopolysaccharides (LPS), a major component of the outer membrane of gram-negative bacteria, are capable of causing a wide variety of pathophysiological effects, such as endotoxic shock and tissue injury, and can be lethal in both humans and animals [3]. Lipopolysaccharides can induce tissue and organ injuries, apart from producing and releasing various pro-inflammatory cytokines and mediators, especially though the increased production of reactive oxygen intermediates such as superoxide radical (O_2_•^−^), lipid peroxides, and nitric oxide, which cause oxidative stress [4]. Thus, the search for effective, nontoxic, natural agents with antioxidant properties that are efficient in inhibiting the production of reactive oxygen radicals is very important for the prevention and treatment of LPS-induced tissue injuries and sepsis.

Supplementation with dietary antioxidants can be helpful to attenuate the damage to the body induced by oxidative stress due to LPS [5], [6], and can be used as potential therapeutic agents or as preventive drugs for the risk of many free radical-mediated diseases [7]. However, there are restrictions on the use of synthetic antioxidants, such as butylated hydroxyanisole (BHA) and butylated hydroxytoluene (BHT), because they may pose a risk to human health [8]. Therefore, efforts to develop alternative antioxidants of natural origin have been reinforced, and numerous studies have been conducted in order to evaluate the antioxidant capacity of certain compounds of plant origin.

The intensity effects of LPS depend on the animal species. Some animals, such as humans, rabbits, and pigs, are very sensitive to low doses of LPS, whereas animals like mice have relatively low sensitivity to LPS [9]. Both mice and rats are commonly used as animal models in clinical studies of oxidative stress in humans. The rodents, however, have limited use as models because their anatomical, physiological, and inflammatory responses, both cellular and humoral, are different from those of humans [10]. Pigs are recognized as an ideal animal model for human health research, based upon their similarity in size, organ development, physiology, disease progression and pathophysiologic responses [11], [12]. Therefore, pigs were chosen as an experimental model for functional food researches and this may be more advantageous than using mice. However, the effect of LPS on oxidative stress in pigs and the protective mechanisms involved in flavonoid therapy have remained limited.

The present study was designed to investigate the *in vitro* and *in vivo* antioxidant capacity of APS. In this study, we evaluated the effects of APS as an antioxidant by using several antioxidant assays *in vitro*, including ferric-reducing antioxidant power (FRAP) assay, scavenging activity on 2,2′-azinobis-(3-ethylbenzthiazoline-6-sulphonate) (ABTS), 1,1-diphenyl-2-picrylhydrazyl (DPPH), hydrogen peroxide (H_2_O_2_), and O_2_•^−^. Also, we evaluated the antioxidative effects of APS on the oxidative stress of pig erythrocytes induced with 2,2′-azobis-2-amidinopropane dihydrochloride (AAPH), a free radical generator, to test the markers of oxidative stress, including hemolysis, lipid peroxidation, superoxide dismutase (SOD) enzyme activity, and phosphatidylserine exposure on the cell surface, to investigate the protective effect of APS on early cellular apoptotic responses to oxidative stress. Finally, we studied the antioxidant potential of APS *in vivo* by evaluating the activities of antioxidant enzymes and lipid peroxidation levels in the LPS-induced piglets.

## Materials and Methods

### Chemicals and reagents

APS was purchased from the Aladdin Industrial Corporation (HPLC purity 95%, Shanghai, China). DPPH, ABTS, phenazine methosulfate (PMS), nicotinamide adenine dinucleotide (NADH), nitroblue tetrazolium (NBT), 2,4,6-tri(2-pyridyl)-s-triazine (TPTZ), AAPH, BHA, and LPS (from Escherichia coli O55:B5) were purchased from Sigma-Aldrich (St. Louis, MO). Commercial kits used for the determination of enzymes activities of SOD, glutathione peroxidase (GSH-Px), catalase (CAT), and total protein contents were purchased from the Nanjing Jiancheng Bioengineering Institute (Jiangsu, China). All other chemicals used were obtained from the Shanghai chemical agents company, China and were of analytical grade.

### DPPH radical scavenging activity

The DPPH radical scavenging activities of APS and BHA were evaluated by the method of Xu *et al*. [13] with minor modifications. Briefly, 2.0 mL of samples in methanol at different concentrations (2, 4, 6, 8, 10 µg/mL) was mixed with aliquots of 0.1 mM DPPH solution in methanol. The mixture was vortexed for 1 min and incubated at ambient temperature for 30 min in the dark, and then the absorbance at 517 nm was measured using a spectrophotometer. The DPPH radical scavenging activity was calculated by the following equation:
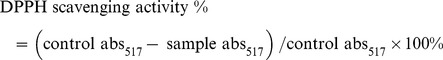



### ABTS^•+^ radical scavenging activity

The ability of antioxidant molecules to quench ABTS radical cation (ABTS^•+^) was determined according to the method of Okamoto *et al*. [14]. A stable stock solution of ABTS^•+^ was produced by the reaction of a 7 mM aqueous solution of ABTS with 2.45 mM potassium persulfate (final concentration) and allowing the mixture to stand in the dark at room temperature for 16 h before use. 1 mL of ABTS^•+^ stock solution was added to the 3 mL of sample solutions at various concentrations (2, 4, 6, 8, 10 µg/mL). The contents were mixed well and incubated at 3°C exactly for 30 min. Then the absorbance was determined at 534 nm. The ABTS^•+^ radical scavenging activity was calculated as follows:
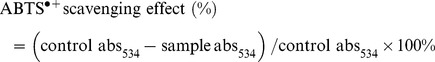



### Ferric - reducing antioxidant power (FRAP) assay

The antioxidant capacity of APS was estimated according to Pulido, *et al*. [15]. 1.5 mL of FRAP reagent, prepared freshly and warmed at 37°C, was mixed with 150 µL of distilled water and 50 µL of test sample solutions at various concentrations (10, 20, 30, 40, 50, 60 µg/mL) or methanol (for the reagent blank). All samples were incubated at 37°C for 30 min in the dark. At the end of incubation, the absorbance readings were taken immediately at 593 nm. The final results were expressed as the concentration of antioxidants having a ferric reducing ability equivalent to that of 1 µM FeSO_4_. A standard curve ranging from 5 to 150 µM of FeSO_4_•7H_2_O was used for calibration.

### H_2_O_2_ scavenging activity

The ability of scavenging H_2_O_2_ was determined according to the method reported by Ak & Gulcin [16]. The reaction mixture was composed of 3.4 mL of sample solutions at various concentrations (10, 20, 30, 40, 50, 60 µg/mL) in phosphate buffer (0.1 M,pH = 7.4), 0.6 mL of H_2_O_2_ solution (0.04 M). Absorbance of reaction mixture at 230 nm was determined after 10 min against a blank contained the phosphate buffer without H_2_O_2_. The percentage of H_2_O_2_ scavenging by sample was calculated using the following equation:
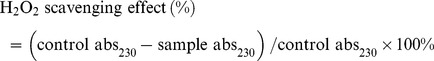



### O_2_•^−^ scavenging activity

The O_2_•^−^ scavenging activity was measured by the method of Yen & Chen [17]. An aliquot of each of the following solutions prepared in 0.1 M phosphate buffer at pH 7.4∶150 µM NBT, 60 µM PMS and 468 µM NADH. 1 mL of each of above-mentioned solutions and 1 mL sample solutions at various concentrations (10, 20, 30, 40, 50, 60 µg/mL) were added respectively, to the reaction mixture, and incubated at room temperature for 5 min. The absorbance was measured at 560 nm. The scavenging activity on O_2_•^−^ was expressed as:
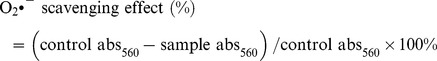



### Protective effect of APS on AAPH-induced hemolysis

Swine blood was collected by venipuncture into tubes containing heparin and centrifuged at 1500×g for 10 min at 4°C. After removal of plasma and buffy coat, erythrocytes were washed three times with cold phosphate buffered saline (PBS). 5% suspension of erythrocytes in PBS were preincubated at 37°C for 30 min in the chosen concentrations of APS (2.5, 5, 10 µM), and then incubated with AAPH (final concentration 75 mM) for 5 h with gentle shaking. Aliquots of the reaction mixture were taken out and centrifuged at each hour of incubation, diluted with saline, and centrifuged at 1500×g for 10 min. The percentage of hemolysis was determined by measuring the absorbance of the supernatant (A) at 540 nm and compared with that of complete hemolysis (B) by treating an aliquot with the same volume of the reaction mixture with distilled water. The hemolysis percentage was calculated using the formula: A/B ×100%. Three independent experiments were used for these calculations.

### Measurement of erythrocyte SOD activity and lipid peroxidation level

The enzyme activity of SOD was based on its ability to inhibit the oxidation of the sample by the xanthine–xanthine oxidase system. One unit of SOD activity is defined as that amount of enzyme required to inhibit the reduction of SOD by 50% under the specified conditions, and the data were expressed as U/mg Hb. The level of lipid peroxidation was indicated by the content of malondialdehyde (MDA) using a thiobarbituric acid reaction (TBAR) method [18]. MDA content was expressed as nmol/mg Hb.

### FACS analysis of annexin V binding

Phosphatidylserine exposure on erythrocytes was performed using the Alexa Fluor 488 Annexin V/Dead cell apoptosis kit (Invitrogen, cat. no V13245) according to the manufacturer’s instruction. FACS analysis was performed as described by Agalakova & Gusev [19]. After the preincubation of APS and AAPH, the erythrocytes were washed twice with cold PBS (pH = 7.4, containing 150 mM NaCl, 1.9 mM Na_2_HPO_4_ and 8.1 mM NaH_2_PO_4_) and resuspended (5% suspension) in annexin-binding buffer. Then, the cells were stained with FITC- annexin V (1∶50) at room temperature in the dark for 15 min. After incubation, samples were finally diluted 1∶ 5 in the same buffer and measured by flow cytometric analysis on a FACS-Calibur from Becton Dickinson (Heidelberg, Germany). The annexin V fluorescence intensity was measured in FL-1 with 488 nm excitation wavelength and 530 nm emission wavelength.

### Assay of antioxidant activity *in vivo*

#### Animals and experimental design

Antioxidant enzymes activities, total antioxidant capacity and lipid peroxidation, *in vivo*, were determined. The animal use protocol for this research was approved by the Animal Care and Use Committee of Nanjing Agricultural University, Nanjing, PR. China. All piglets in this experiment were crossbred (Duroc×Landrace×Yorkshire) with an initial body weight of 7.5–8.3 kg and weaned at 4 weeks old. After weaning, piglets were housed in clean stainless steel metabolic cages in an ambient temperature of 27±1°C with a 12 h light/dark sequence. Each pen had a feeder and a nipple water to allow piglet *ad libitum* access to the basal diet and water. A total of 16 female piglet were randomly divided into the following four groups: the control group, the animals fed the basal diet for 27 days and receiving intraperitoneal (i.p.) administration of sterile saline on 28^th^ day; LPS group, the animals were administered the basal diet for 27 days and were challenged with LPS (25 µg/kg) i.p. on 28^th^ day; APS+LPS groups, the animals were treated with APS (100 and 400 mg/kg; as-fed basis) p.o. for 27 days, and received LPS (25 µg/kg) on the 28^th^ day.

### Measurement of SOD, GSH-Px and CAT activities in lung and liver of piglet

After 6 h of LPS injection, all the animals were sacrificed. Their lung and liver were removed rapidly, weighed and homogenized in ice-cold physiological saline. Then, the homogenate was centrifuged at 3500 g at 4°C for 15 min to remove cellular debris, and the supernatant was collected for analysis. The determination of the activity of SOD, CAT, and GSH-Px were described by Ahmad *et al*. [20] and measured according to the instructions in the kits. Protein contents were determined by a previously reported method [21].

### Measurement of T-AOC in serum, MDA level and carbonyl content in lung and liver of piglet

Blood Samples were collected into plastic tubes (Ganda, Jiangxi China) containing no anticoagulant. Blood samples were allowed to clot at room temperature and were centrifuged at 4000 g for 10 min at 4°C to obtain the required serums. The total antioxidant capability (T-AOC) [20], MDA, and protein carbonyl content were measured according to the instructions in the kits.

### Statistical analysis

The experimental results are expressed as mean ± standard error of means (SEM). SPSS (version 16.0) was used for statistical analysis. Data on DPPH, ABTS^•+^, O_2_•^−^, H_2_O_2_, and FRAP were analyzed by two-way ANOVA to determine the main effects of antioxidant (AO) and concentration (CT) and their interaction, followed by unpaired *t*-test for comparisons of two groups at each content point. Data on erythrocyte and piglet were analyzed by one-way ANOVA supplemented with Tukey’s HSD post hoc test for multiple comparisons between groups. *P*<0.05 was considered significant.

## Results

### DPPH radical scavenging activity

**Figure 1:**
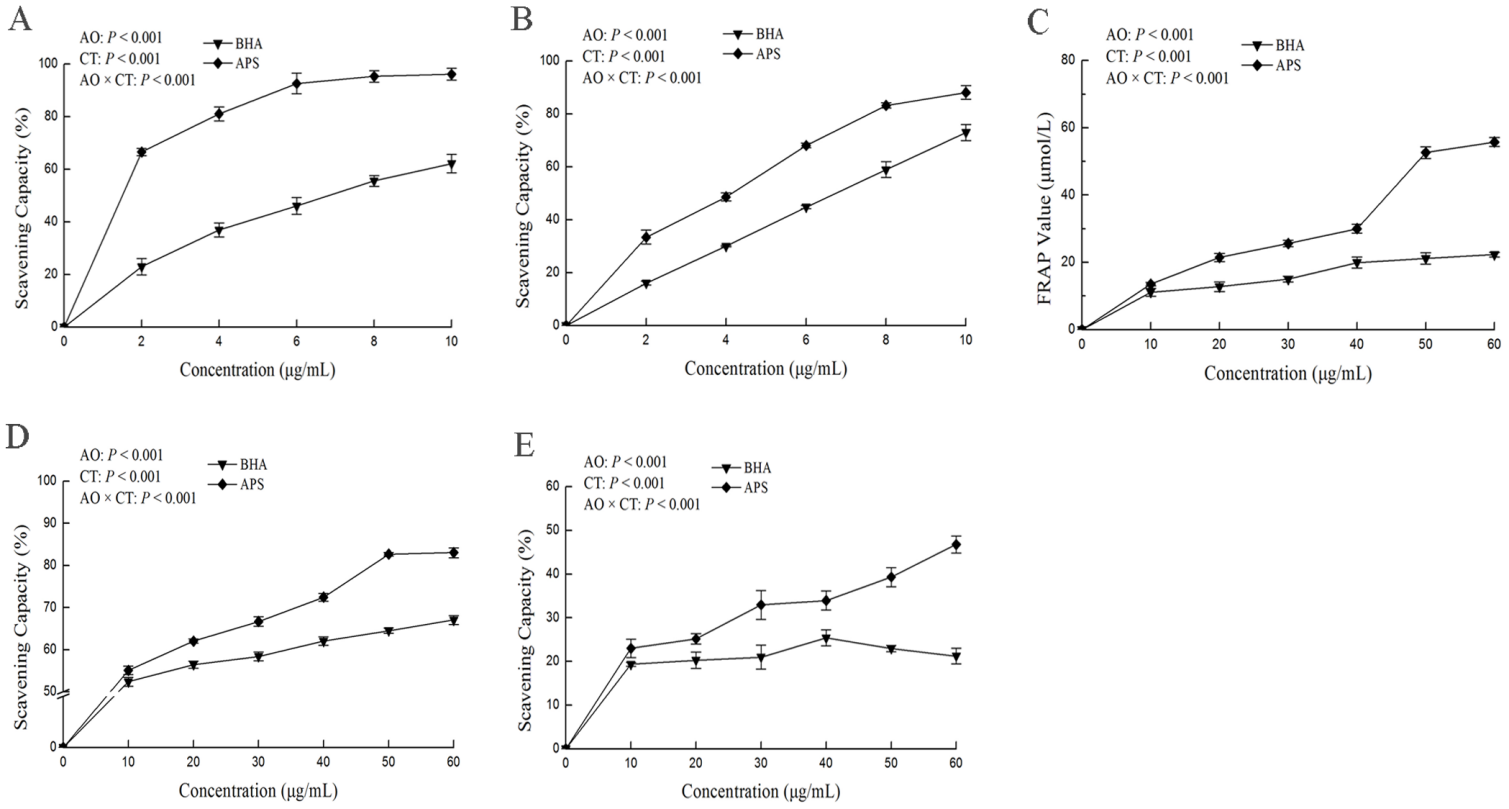
DPPH scavenging activity (A),ABTS scavenging activity (B),FRAP assay (C),H_2_O_2_ scavenging activity (D) and O_2_•^−^scavenging activity (E) of APS and BHA. Data are expressed as the mean ± SEM of three parallel experiments. The *P* value showed the effects of antioxidant (AO) and concentration (CT), and their interaction on radicals scavenging activity.

The scavenging DPPH radical ability of APS and the synthetic antioxidant BHA is shown in Figure 1A. At concentrations of 2 to 10 µg/mL, the scavenging activities of APS were 66.55% to 96.19%, while the scavenging activities of BHA were 22.96% to 62.18%. The results showed that APS is more efficient (*P*<0.05) in the scavenging DPPH radical ability than the BHA.

### ABTS^•+^ radical scavenging activity

The ABTS^•+^ radical scavenging activities of APS and BHA were positively correlated with their concentrations (Figure 1B). The scavenging activity of APS was significantly higher (*P*<0.05) than that of BHA. At the concentration of 10 µg/mL, the scavenging activities of APS and BHA were 83.13% and 73.02%, respectively.

### FRAP assay

The FRAP values of APS were increased in a concentration-dependent manner (Figure 1C). At a concentration of 60 µg/mL, the reducing power of APS and BHA was 55.80 and 22.30 µM FeSO_4_, respectively. The APS had greater (*P*<0.05) reducing capacity than the reference control of BHA.

### H_2_O_2_ scavenging activity

Figure 1D depicts the scavenging capacity of APS against H_2_O_2_. The concentration range (10 to 60 µg/mL) of APS was capable of scavenging H_2_O_2_ in a concentration-dependent manner. Further, when compared with BHA, the APS was more effective (*P*<0.05) for scavenging H_2_O_2_. The H_2_O_2_ scavenging activity of APS was 83.05% at a concentration of 60 µg/mL, while it was 67.05% for BHA.

### O_2_•^−^ scavenging activity

Figure 1E depicts the scavenging capacity of APS against O_2_•^−^. Increasing the concentrations of APS increased its O_2_•^−^ scavenging capacities. However, the O_2_•^−^ scavenging capacities of BHA was increased from 10 to 40 µg/mL, and then decreased at higher concentrations. At level of 60 µg/mL, the scavenging activities of APS and BHA were 46.78% and 21.25%, respectively. These results showed that the APS was more efficient (*P*<0.05) than BHA antioxidant.

### Effect of APS on AAPH-induced erythrocytes hemolysis

**Figure 2:**
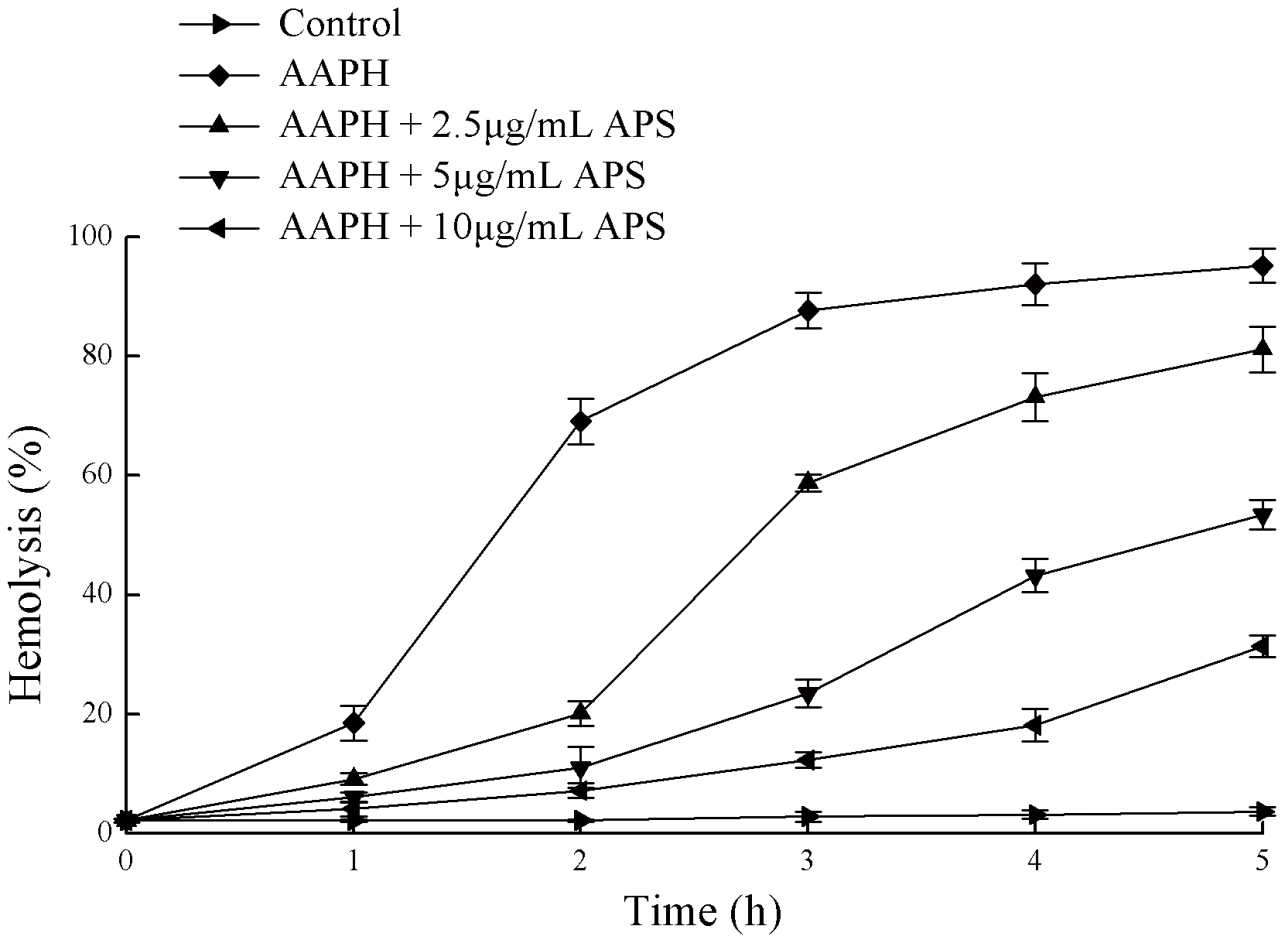
Effect of APS on AAPH-induced erythrocytes hemolysis. The erythrocytes were preincubated with APS at the indicated concentrations for 30 min at 37°C. The cell suspension was then incubated with 75 mM AAPH for 5 h at 37°C. Data are expressed as the mean ± SEM of three parallel experiments.

The AAPH-induced hemolysis in pig erythrocytes and the protective effect of the APS at different concentrations (2.5, 5, 10 µg/mL) are shown in Figure 2. Erythrocytes incubated at 37°C as a 5% suspension in PBS remained stable, with little hemolysis within 5 h (3.72% ±0.11). When AAPH was added to the erythrocyte suspension, after a relatively flat growth, hemolysis significantly increased (*P*<0.05) from 18.54±2.87% (1 h) to 87.66±2.14% (2 h), and it also increased in a time-dependent manner. After 5 h of incubation, the hemolytic activities of APS at different concentrations (2.5, 5, 10 µg/mL) against AAPH were 81.12±2.75%, 53.46±1.74%, and 31.38±1.55%, respectively.

### Effect of APS on AAPH-induced lipid peroxidation in erythrocytes

**Figure 3:**
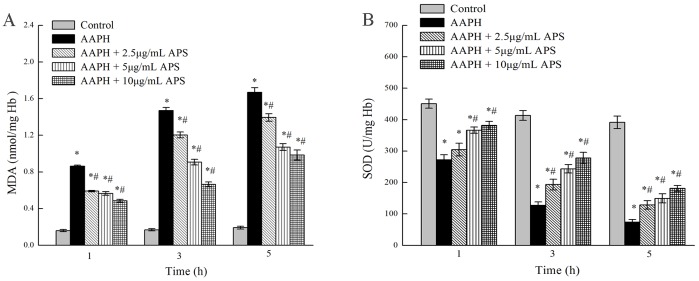
Effect of APS on MDA level (A) and SOD activity (B) in erythrocytes induced by AAPH. The pig erythrocytes were preincubated with APS at the indicated concentrations for 30 min at 37°C. The cell suspension was then incubated with 75 mM AAPH for 1, 3, 5 h at 37°C. Data are expressed as the mean ± SEM of three parallel experiments. *indicates a significant difference from control group (*P*<0.05); ^#^ indicates a significant difference from AAPH group (*P*<0.05).

The MDA level in the pig erythrocytes of the control group were stable (0.191±0.016 nmol/mg Hb) after 5 h of incubation (Figure 3A). The MDA content was significantly increased (*P*<0.05) by 443.6%, 774.3%, and 875.4%, at 1, 3, and 5 h after incubation, respectively, with 75 mM AAPH as compared to the control samples. However, pretreatment with the APS concentrations (2.5, 5, 10 µg/mL) significantly decreased (*P*<0.05) the MDA levels compared to the AAPH group. After 5 h of incubation, in AAPH added erythrocytes, the MDA levels were significantly decreased (*P*<0.05) by 16.4%, 35.7% and 40.9% after addition of APS concentrations of 2.5, 5 and 10 µg/mL, respectively.

### Effect of APS on AAPH-induced SOD in erythrocytes

Activity of SOD enzyme was significantly decreased (*P*<0.05) in a time-dependent manner after the exposure to AAPH, as compared with control group (Figure 3B). However, after 5 h of incubation, the APS (2.5, 5, 10 µg/mL) pretreated erythrocytes showed a significant (*P*<0.05) protective action against the decrease of SOD enzyme activity induced by AAPH.

### Annexin V binding

**Figure 4:**
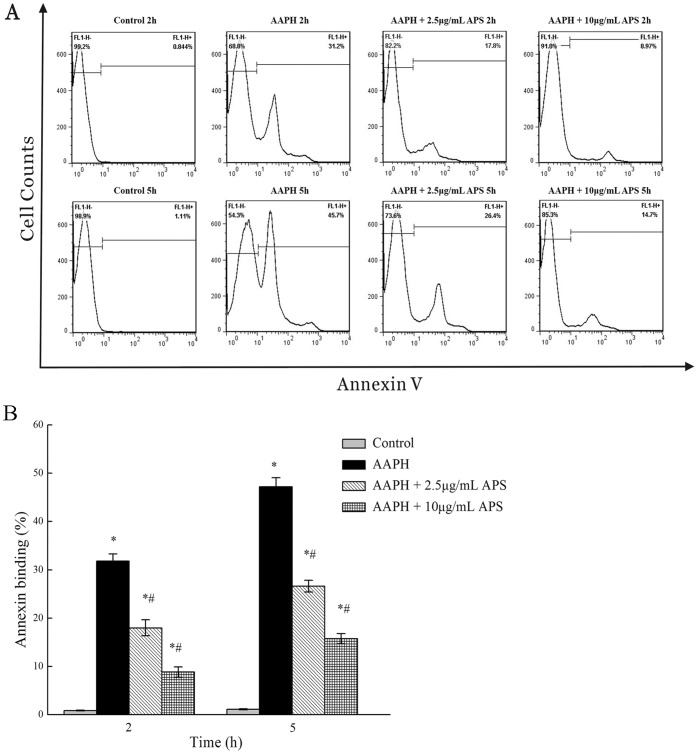
Effect of APS on annexin binding of erythrocytes following AAPH. A Representative original flow cytometry histograms of annexin binding of erythrocytes incubated in APS (2.5 and 10 µg/mL) or 75 mM AAPH for 2, 5 h. B Representative mean ± SEM of erythrocyte annexin binding after a 2, 5 h exposure to AAPH with and without APS. *indicates a significant difference from control group (*P*<0.05); ^#^ indicates a significant difference from AAPH group (*P*<0.05).

As shown in the representative dot plot (Figure 4A and 4B), the percentage of annexin-positive erythrocytes from AAPH exposure was significantly higher (*P*<0.05) by 31.8±1.5% than in the APS (2.5 and 10 µg/mL) preincubated groups (18.0±1.7% and 8.8±1.1%), respectively, at 2 h of incubation. After 5 h, the percentage of annexin-binding erythrocytes was significantly increased (*P*<0.05) by 47.2±1.9%, while in the APS (2.5 and 10 µg/mL) pretreated groups, the percentage of annexin-binding erythrocytes was 26.60±1.2% and 15.7±1.1%, respectively.

### Effects of APS on serum T-AOC level in LPS-induced piglet

The LPS-treated piglets showed significantly decreased (*P*<0.05) T-AOC levels in the serum as compared with the control piglets (Table 1). The serum T-AOC significantly increased (*P*<0.05) after the supplementation of dietary APS (400 mg/kg) compared with the non-APS-treated LPS injected group.

**Table 1:** Effect of APS on activities of T-AOC, SOD, GSH-Px and CAT, levels of MDA and contents of protein carbonyl in LPS-induced piglets.

Parameters	control group	LPS group	LPS+APS (100 mg/kg)	LPS+APS (400 mg/kg)
Serum				
T-AOC	33.95±4.58^a^	17.08±2.04^c^	21.76±2.59^bc^	24.12±2.76^b^
Liver				
SOD	353.85±9.63^a^	243.85±8.91^c^	262.67±16.18^c^	295.51±12.61^b^
GSH-Px	38.14±4.34^a^	17.12±7.57^b^	26.64±4.97^a^	29.92±4.76^a^
CAT	27.20±4.61^a^	12.78±2.05^b^	14.36±1.05^b^	15.44±2.78^b^
MDA	2.56±0.31^c^	5.42±0.55^a^	4.58±0.70^ab^	3.70±0.54^bc^
Protein carbonyl	1.12±0.28^b^	3.81±0.84^a^	2.63±1.19^ab^	2.39±0.98^ab^
Lung				
SOD	163.63±31.31^a^	70.39±7.17^b^	100.08±16.8^b^	106.49±19.28^b^
GSH-Px	7.55±1.08^a^	4.37±0.68^b^	5.38±0.94^ab^	6.15±1.64^ab^
CAT	18.36±4.17^a^	11.73±1.61^b^	13.30±1.43^ab^	13.79±1.60^ab^
MDA	1.61±0.15^b^	3.35±0.44^a^	2.88±0.18^a^	2.79±0.12^a^
Protein carbonyl	0.27±0.03^b^	0.46±0.06^a^	0.35±0.05^ab^	0.32±0.07^ab^

Data on T-AOC are expressed as U/mL. Data on SOD, GSH-Px and CAT are expressed as U/mg protein. Values of MDA are expressed in nmol/mg protein. Protein carbonyl content expressed as µmol/mg protein. Data were presented as mean ± SEM (n = 4) and evaluated by one-way ANOVA followed by Tukey’s test. Values in a row not sharing same superscript letter are significantly different, *P*<0.05.

### Effect of APS on antioxidant enzyme activities in LPS-induced piglet

The activities of antioxidant enzymes GSH-Px, SOD, and CAT in tissue homogenates of lung and liver in the present study are shown in Table 1. The LPS-challenged piglets showed a significant decrease (*P*<0.05) in the activities of antioxidant enzymes as compared with the control group pigs. Administration of APS increased LPS-induced depletion of antioxidant enzyme activities both in the liver and lung of piglets. The level of 400 mg/kg APS to LPS-treated piglets increased (*P*<0.05) the activities of hepatic SOD and GSH-Px. However, 100 mg/kg dietary APS to LPS-treated piglets increased the SOD, GSH-Px, and CAT enzyme activities both in the liver and lung, but this was not statistically significant (*P*>0.05) in LPS-stressed piglets.

### Effect of APS on lipid peroxidation and protein carbonyl in LPS-induced piglet

The results of present study showed a significant increased (*P*<0.05) in MDA and protein carbonyl contents in LPS-stressed piglets as compared with the control (Table 1). In piglets, compared with the LPS-injected group, the supplementation of dietary APS (100 and 400 mg/kg) decreased the MDA levels by 15.5% (*P*>0.05) and 31.7% (*P*<0.05) respectively in the liver; however, in the lungs the decreased was by 14.0% and 16.7% (*P*>0.05), respectively. The protein carbonyl contents of liver and lung were significantly higher (*P*<0.05) in LPS-injected group than that of the control group, while the dietary APS supplementation (100 and 400 mg/kg) had no significant effect on the protein carbonyl contents of liver and lung in both the control and LPS- injected pigs.

## Discussion

In recent years, there has been a great deal of attention towards the field of antioxidants. Food scientists are interested in antioxidants because they can prevent rancidity in diets. Antioxidants have become of interest to biologists and clinicians because they can protect the body from oxidative damage. Ampelopsin is a major flavonoid, which has a close relationship with various kinds of biological activities and antioxidant capacity. In our experiments APS was analyzed using both chemical and biological assays.

### DPPH and ABTS^•+^ radical scavenging activity

The DPPH assay is based on the reduction of DPPH•, a stable free radical that accepts an electron or hydrogen radical to become a stable diamagnetic molecule and is reduced to DPPHH, resulting in decolorization (yellow color) with respect to the number of electrons captured. Thus, it can be widely used as a model to study the reaction rates of antioxidants in order to quantify and compare the scavenging ability of various compounds [22]. Another common organic radical that has been applied to determine the antioxidant activity of both lipophilic and hydrophilic compounds is ABTS radical cation [23]. When estimating the ABTS^•+^ radical scavenging activities of BHA, similar values were obtained from the DPPH assays, suggesting that these two assays were positively correlated [24]. It was found that APS had a much higher antioxidant capacity in the DPPH assay than in the ABTS assay, possibly due to the difference in the molecular structures of the two radicals and in the chemical reactions.

### FRAP assay

On the basis of previous reports, it is clear that there is an association between the antioxidant capacities of antioxidants and their reducing power [25]. We evaluated the reducing power of APS using FRAP assay. The FRAP method is based on the reducing potential of the antioxidant to convert Fe^3+^ to Fe^2+^ in the presence of TPTZ, producing an intense blue-colored Fe^2+^–TPTZ complex with an absorption maximum of 593 nm.

The DPPH, ABTS, and FRAP assays belong to an electron transfer mechanism and they are based on measurements of the capacities of antioxidants in the reduction of colored oxidants. The ABTS/DPPH methods measure the active compound capacity against an oxidant, while the FRAP assay directly measures the reducing capacity of substances, which is an important parameter for a compound to be considered a good antioxidant.

### H_2_O_2_ and O_2_•^−^ scavenging activity

Reactive oxygen species (ROS), such as O_2_•^−^, H_2_O_2_ and hydroxyl radical (OH•), can act as signaling intermediates. However, oxidative stress occurs as a result of higher ROS levels due to an imbalance between ROS production and endogenous antioxidant defense systems [26]. Both DPPH and ABTS represent limited oxidation situations because they do not exist *in vivo*. Therefore, we also investigated the scavenging activity of APS against H_2_O_2_ and O_2_•^−^.

In contrast, in the more electrophilic forms of ROS, H_2_O_2_ is a small, stable, uncharged, and freely diffusible molecule that can be rapidly synthesized and destroyed in response to external stimuli [27]. Although H_2_O_2_ is not very reactive, its high penetrability in cellular membranes leads to OH• formation after its reaction with Fe^2+^ or the O_2_•^−^ in the cells [28], [29]. Thus, removing H_2_O_2_ is very important for living organisms to avoid damage. O_2_•^−^ is a toxic species and forms first in numerous biological reactions among different ROS *in vivo* [30]. Although O_2_•^−^ cannot directly initiate lipid oxidation, it can act as a potential precursor to generate more reactive radical species, such as OH• and peroxynitrite, which interact with lipids, proteins, and DNA molecules, eventually leading to various chronic diseases [31]. Therefore, measuring the potential of compounds to scavenge O_2_•^−^ is one of the most important methods of clarifying the mechanism of antioxidant activity. The results of our present study suggested that the APS could be considered a good scavenger of H_2_O_2_ and O_2_•^−^.

### Effect of APS on AAPH-induced erythrocytes hemolysis

In this study, to confirm the antioxidant capacity of APS, we also evaluated its antioxidant potential by considering its efficacy in inhibiting AAPH-induced oxidative stress in pig erythrocytes. Erythrocytes have a well-established antioxidant defense system; even though, they are more susceptible to oxidation due to the presence of high polyunsaturated fatty acid (PUFA) content in membranes, the O_2_ transport associated with redox active hemoglobin molecules, and transition metals such as iron and copper. Hemolysis of pig erythrocytes is a very good model for studying free radical induced oxidative damage to membranes, and for evaluating the antioxidant activity of APS. Erythrocytes are the most abundant cells in humans and animals and can easily become susceptible to oxidative damage with blood flow, usually resulting from exposure to drug side effects, toxic chemicals, and transition metal excesses. Excessive ROS continuously produced in erythrocytes due to these internal and external factors then overwhelmed cellular defenses, leading to protein oxidation and lipid peroxidation, which results in hemolysis and ultimately in cell death.

Erythrocytes have several enzymatic and non-enzymatic cellular antioxidant defenses such as SOD, glutathione (GSH), and ubiquinone. The changes in these biological mechanisms may be responsible for the previous lag phase shown in AAPH-induced hemolysis. Furthermore, APS remarkably delayed AAPH-induced hemolysis and had a dose-dependent correlation. Flavonoids can directly scavenge free radicals, and certain flavonoids also have the ability to incorporate into the hydrophobic core of the bilayer membranes. These changes in their fluidity and stability could strictly limit the diffusion of free radicals, thus decreasing the oxidative process throughout the membrane [32]. Therefore, in the present study, preincubation of the APS before AAPH addition, was considered as the interaction of an antioxidant with the cell membrane of erythrocytes. It was also found that the hemolysis rate inhibited by the APS slowed after the intense attack of peroxyl radicals generated by AAPH. The reason may be that the end products after the oxidation of flavonoids also exhibit antioxidant properties [33]. At the lower APS concentrations, oxidation products can still play the role of antioxidants, thus decreasing the hemolysis rate. All the results of our present study indicated that the APS could efficiently protect erythrocytes against AAPH-induced hemolysis. Our results are also consistent with the findings of previous studies [34]–[36] and indicated that the different flavonoids exhibit excellent abilities to protect against AAPH-induced hemolysis in erythrocytes [34]–[36].

### Effect of APS on AAPH-induced lipid peroxidation in erythrocytes

In biological systems, lipid peroxidation can reduce membrane fluidity, influence enzymatic activities, inactivate membrane-bound proteins, and break down into cytotoxic aldehydes such as MDA or hydroxynonenal. The MDA, an indicator, is a main product of lipid peroxidation. A higher MDA level suggests that there is more lipid peroxidation and high oxidant stress [37].

Hemolysis caused by radicals can be characterized mainly by two key events: lipid peroxidation and redistribution of oxidized band 3 protein within the membrane [38]. Exposure of erythrocytes to free radicals generates oxidants such as AAPH and PUFA that can be converted into unstable lipid hydroperoxides. Lipid peroxidation continues until the formation of MDA, which can alter the proteins and membrane lipids together, and ultimately leads to the death of cells. The specific protein-lipid interaction provides a mechanism for the formation and stabilization of membrane domains and band 3 protein, a major erythrocyte transmembrane protein that interacts with many phospholipids at its surface [39]. The MDA accumulation can affect the anion transport and functions of the band 3 associated enzymes such as glyceraldehyde-3-phosphate dehydrogenase and phosphofructokinase. This may be the possible reason for the higher MDA content in AAPH-added erythrocyte hemolysate at longer durations.

### Effect of APS on AAPH-induced SOD in erythrocytes

The SOD, the first line of defense against oxygen-derived free radicals, catalyze the dismutation of O_2_•^−^ into O_2_ and H_2_O_2_ [20]. Due to the lack of mitochondria, cytoplasmic SOD have key roles in erythrocytes [40]. Our results are in agreement with the previous study by Alvarez-Suarez [41] who reported that the flavonoid quercetin protected against the decrease of SOD activity in erythrocytes induced by AAPH.

Erythrocytes are well protected against ROS by abundant Cu/Zn-SOD, which scavenges free radicals to prevent the formation of methemoglobin. Erythrocyte damage due to oxidative stress is generally thought to be the result of two processes: the oxidation of hemoglobin, followed by the conversion of metHb to hemichromes; and the free radicals attack on membrane components, including the PUFA side chains of the membrane lipids, reduced thiol groups, and other susceptible amino acid chains of membrane proteins [42]. In our present study, the results showed that the APS had a protective action against the formation of MDA. Moreover, the addition of APS in AAPH- induced erythrocytes caused an increase in SOD enzyme activity compared to the AAPH group without APS-treated erythrocytes, possibly linked to the ability of APS to interact with O_2_•^−^ and subsequently scavenge them. Further experiments are required to understand the exact molecular mechanism.

### Annexin V binding

We further investigated the protective role of APS in pig erythrocytes using annexin V-FITC for the phosphatidylserine (PS) exposure analysis, one of the markers used for cell death or suicidal erythrocyte death (eryptosis) [43]. Oxidative stress, energy depletion, or lower efficiency of antioxidant defense system [43], [44], enhance Ca^2+^ entry via the cation channels, leading to higher intracellular Ca^2+^ concentrations. Erythrocyte PS exposure is accomplished by cell membrane scrambling, which is triggered by an increase in cytosolic Ca^2+^ activity. The exposure of PS at the cell surface favors binding to the respective PS receptors expressed by macrophages [45]; therefore, erythrocytes with PS treatment are more rapidly cleared than non-PS-treated pig erythrocytes. The present study results suggest that the APS prolongs erythrocyte exposure to oxidative stress by increasing the inhibition of apoptotic cellular responses.

### Antioxidant effects of APS in LPS-induced piglet

It is well known that the direct toxic effect of LPS in a variety of organs is to increase the formation of reactive oxygen intermediates such as O_2_•^−^, peroxides, and nitric oxide, especially their secondary products, such as MDA, which is an index of oxidative stress, and these increase in a time- and dose-dependent manner [46]. Therefore, the injection of LPS can be a good model for the simulation of oxidative stress in experimental animals. Among the various organs susceptible to LPS, lungs are most commonly affected, which presents as an acute lung injury or, in its extreme manifestation, as acute respiratory distress syndrome. Oxidative stress induced by LPS rapidly affects other organs, such as the liver, which plays a vital role in the detoxification of LPS. Kupffer cells, the resident macrophages of the liver, are the major targets of LPS, and produce excessive amounts of O_2_•^−^ on activation by LPS stress [47]. In addition, LPS stimulation induces polymorphonuclear neutrophil activation and migration into the liver [48], which is another source of free radicals. The *in vitro* antioxidant assays (Figure 1) results showed that the APS could reduce LPS-induced oxidative stress in piglets, especially in organs such as lung and liver.

All organisms have protective systems of antioxidant defense to maintain the cellular redox state, including both antioxidant enzymes and non-enzymatic antioxidants, in order to minimize or even prevent the injuries to organs and body systems caused by ROS. On the other hand, blood is the first stage of LPS detoxification, and measurements of blood biochemical markers have key clinical importance in the diagnosis of malfunctioning of body organs. The serum T-AOC level reflects the total antioxidant status of the body. The results of our present study indicated that APS may increase resistance to oxidative stress by enhancing T-AOC. Our results are in agreement with the previous reports indicating that the different flavonoid-rich extracts have the ability to decrease oxidative stress by promoting T-AOC [49], [50].

The three main antioxidant enzymes, GSH-Px, SOD, and CAT, are the major antioxidant enzymes in the antioxidant system, which can help protect the body against oxidative damage [40]. SOD catalyze the production of O_2_ and H_2_O_2_ from O_2_•^−^, which in turn are decomposed to water and oxygen by GSH-Px and CAT enzymes, thus preventing the formation of OH•. Several studies have reported that the antioxidant capacities were decreased in the liver of LPS-induced animal models [46], [51]. Previous studies have demonstrated that some natural compounds such as curcumin [46], hesperidin [52] and resveratrol [53] increased antioxidant enzyme activities under LPS-induced oxidative stress, but no studies have been done using pig models.

Oxidative damage to proteins, as characterized by the formation of carbonyl groups, is also a highly damaging event, and can occur in the absence of lipid peroxidation [54]. We also investigated the oxidative damage in lipids and proteins. *In vitro* experiments (Figure 3) indicated that the APS could reduce MDA levels in oxidative stress in pig erythrocytes. The present results also suggested that the supplementation of dietary APS was effective in preventing the deleterious consequences of oxidative stress. The results of the present study have been confirmed by other researchers, who showed that supplementation with natural antioxidants or plant extracts exerts protective effects against LPS-induced injuries *in vivo* [55], [56]. The results suggested that the APS had potent antioxidant activities not only via directly scavenging hydroxyl, superoxide, and metal-induced radicals in various radical-scavenging assays, such as the scavenging activities of free radicals, but also indirectly by enhancing antioxidant enzymes and decreasing lipid peroxidation.

In conclusion, the results of our present study clearly indicated that APS had excellent antioxidant capacity *in vitro*. Dietary APS supplementation can provide protection against LPS-induced oxidative injuries in lung and liver tissues of piglets. These findings reveal a new protective mechanism of APS as a potent antioxidant. The results demonstrated that APS is an accessible source of natural antioxidants and can be used in food and as a therapeutic agent. However, further studies are still required to elucidate the molecular mechanisms underlying the protective effects of APS as an antioxidant.
